# Autoamputation of Genitalia in Bipolar Patient

**DOI:** 10.1155/2017/7275816

**Published:** 2017-12-11

**Authors:** Vinod Sharma, Aditi Sharma

**Affiliations:** Department of Psychiatry, Texas Tech University Health Sciences Center, 3601 4th Street, Stop 8103, Lubbock, TX 79430, USA

## Abstract

According to literature, genital self-mutilation (GSM) is more commonly associated with psychosis as compared with self-mutilation as a whole. There have been many case reports of GSM in psychotic disorders. We describe herein a case of a Caucasian, employed, and married male suffering from bipolar disorder type II with history of self-mutilating behavior, who amputated his penis during symptom-free phase of his illness. Several features are reflected as risky elements for genital self-mutilation, for example, homosexual and transsexual tendencies, abandonment of the male genitals, lack of competent male for identification during childhood, feeling of guilt for sexual offences, and self-injuries in anamnesis. This report will highlight various factors responsible for self-mutilation in nonpsychotic and nondelusional person.

## 1. Introduction

Any deliberate destruction or alteration of body tissue in the absence of conscious suicidal intention is defined as self-mutilation, self-injuring, or self-harming behavior [1]. The spectrum of genital self-mutilation (GSM) diverges from a simple skin laceration to total castration of the testis and amputation of penis as seen in this patient. GSM is a rare phenomenon which is increasing in incidence, the reason for which is not clear. The first genital mutilation case was published in 1846. So far about 123 cases, involving both males and females, were identified and reported in literature. The related psychiatric illnesses vary across gender. In females, GSM is mostly associated with personality disorders (predominantly borderline type), whereas in males, psychosis is present in up to 80% of cases [2]. The most common form of self-harming behavior is skin cutting which is more prevalent in females. In males, acts of genital self-mutilation are more and can cause serious damage to sexual and urinary functions [3]. Self-mutilating behavior has been studied in a variety of racial, chronological, ethnic, gender, and socioeconomic populations [4]. The vast majority of reported incidents occurred among single, white male in the age range of 1920s and 1930s [5] and our patient fulfilled this criteria except being married (lives alone).

## 2. Case Description

A 37-year-old married Caucasian male with history of self-mutilating behavior brought to the emergency room followed by penile amputation. The patient invited a random male via Internet for sex and claimed that man hit him on the head after which he lost consciousness. Upon regaining consciousness, his penis was amputated and he was surrounded with blood. On physical examination, multiple healed self-inflicted lacerations on his chest, abdomen, and groin area were seen. After wound closure and perineal urethrostomy, patient was sent to medical floor where he was found putting his finger in the stoma resulting in wound infection for which intravenous and topical antibiotics were started. Several years ago, patient was diagnosed with bipolar disorder II with multiple admissions to psychiatry hospitals due to suicidal ideation. He had tried various mood stabilizers with suboptimal benefits on outpatient (OP) basis and was on quetiapine 300 mg at bedtime prior to this hospitalization. Urology team requested psychiatry consult to evaluate the patient. He admitted that he has always been attracted towards males but could not share feelings with his wife and/or 16-year-old daughter. Since he had moved out of the house for job he had developed “sexual fantasies” towards young males and was feeling guilty and ashamed for having such thoughts. On different occasions, to “punish” him he castrated his left testicle, stabbed penis, and stabbed his abdomen that needed surgical attention. OP medical records showed that he was “plunging himself on knife” to get sexual gratification, and therapy was recommended which patient could not afford. During adolescence, patient was molested by his friend's uncle multiple times. His father was not around and was raised by his mother. During admission and psychiatry evaluation, he reported depressed mood, low energy, poor concentration, guilt feelings, anhedonia, and poor self-esteem for the past several months; however, he denied any suicide ideations or intent or plan. A sitter had to be arranged to keep him from picking his wounds. Patient also stated that he “can get real high and spend a lot of money” when he is in really good mood but has not had these symptoms “in a long time.” He had been to drug rehabilitation programs due to prescription narcotic and benzodiazepine abuse, but he denied current illicit substance abuse. On mental status examination, he appeared to be fairly groomed with normal psychomotor activity and minimal eye contact. Speech was slow in rate and rhythm and normal in tone and volume. Mood was euthymic, and effect was indifferent to his injuries. Thought process was goal-directed. No sensory hallucinations were reported. Thought content is devoid of suicidal or homicidal ideations or intent or plan or any delusions. Insight and judgment were considered to be impaired. He had fair to poor impulse control and was oriented X 3. We diagnosed him with borderline personality disorder and bipolar II disorder, most recent episode depressed. Quetiapine 300 mg at bedtime was resumed for mood stabilization and recommended to follow-up with urologist, psychiatrist, and therapist in OP setting. After 1 week of stay in hospital, he got discharged under stable conditions.

## 3. Discussion and Conclusion

**Figure 1:**
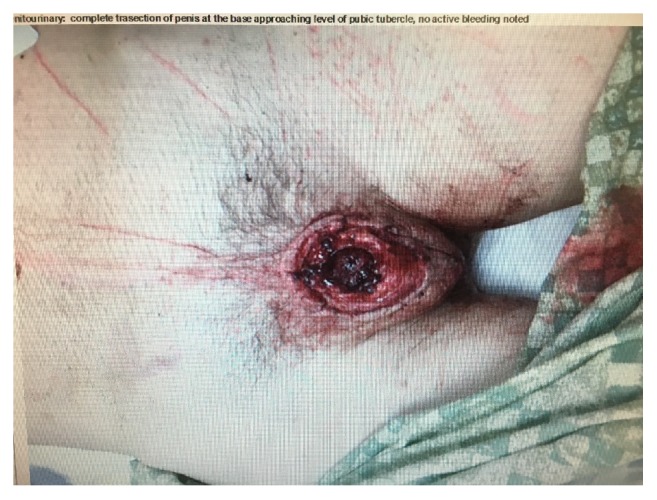
Amputated genitalia with cutting marks on abdomen and groin.

The foremost psychotic impetuses for GSM include delusions often religious and command hallucinations that are seen in paranoid schizophrenia and affective psychosis. Other predisposing factors of GSM include severe deprivation in childhood, pathological feelings of guilt associated with aberrant sexual conduct and conflicts, transvestites, suicide attempts or other self-destructive behaviors in the history, social withdrawal, and being depressed [4, 5]. Mago indicated that the most important factors in the act of psychotic self-mutilation were guilt feeling associated with the sexual conflicts and the religious psychotic experiences. In nonpsychotics, disturbance of sexual identity was the most common cause of genital mutilation [4]. Also self-mutilators with sexual guilt feelings were likely to mutilate themselves more severely than those without [6]. Ozan et al. elucidated the grievous nature of amputation of the penis and it is considered as a suicide attempt in Chinese culture [7]. Literature explicated that the role of psychotropic drugs as a cause of GSM cannot be ruled out in a nonpsychotic person. There have been reports related to use of illicit drugs like cannabis, benzodiazepines, glue sniffing, hallucinogens, amphetamines, and alcohol to be associated with deliberate self-harm [8]. However, drug abuse not likely to have played a significant role in this patient as history repudiated these factors. Direct motives of the act in this index case were guilt feelings associated with his sexual orientation and sexual fantasies. Unlike previous published reports that showed 80% association of automutilation with psychosis [4], this patient did not have any psychotic symptoms and have always been showed up with depressive symptoms during the current and follow-up visits. He denied that autoamputation of penis was an attempt to expiate for past sins or an attempt to change his sex. He claimed that he was not aware of the act of autoamputation and somebody else might have done it. However, the possibility of self-inflicted amputation of penis is much higher given his prior history of recurrent GSM behavior, self-cutting behavior as evident by healed marks in the groin area, nipples (Figure 1). Also, absence of overt trauma to the head, normal head CT, and lack of remorse emphasize that amputation of the penis is self-inflicted. He accepted that he has guilt feelings regarding his sexual orientation and aberrant sexual fantasies regarding young males. It is clear that there is no suicidal intent in the patient. In this patient, we therefore find these features that can be reflected as risk factors for his GSM behavior: (1) absence of father in childhood; (2) childhood sexual abuse; (3) previous self-injuries and self-castration; (4) sexual aberration and fantasies with accompanying guilt feelings; (5) history of substance abuse and bipolar disorder.

This case is unique as at the time of the autoamputation the patient did not have any psychotic features. His disease was well controlled and he had no delusions or religious beliefs as in “Klingsor syndrome” that is an eponym coined to denote GSM resulting from hyperreligious delusion [9]. This case elucidates some of the aspects of the relationship between psychopathology and GSM; still further research in this area is needed to completely explicate the relationship between the two.
